# Female–female aggression in the Gila monster (*Heloderma suspectum*)

**DOI:** 10.1098/rsos.221466

**Published:** 2023-05-10

**Authors:** Gordon W. Schuett, Karl H. Peterson, Anthony R. Powell, John D. Taylor, Jennifer R. Alexander, A. Kristopher Lappin

**Affiliations:** ^1^ Department of Biology | Neuroscience Institute, Georgia State University, Atlanta, GA, USA; ^2^ Chiricahua Desert Museum, Rodeo, NM, USA; ^3^ Department of Herpetology, Houston Zoo, Houston, TX, USA; ^4^ Biological Sciences Department, California State Polytechnic University, Pomona, CA, USA

**Keywords:** Helodermatidae, lizard, ethogram, fighting, bite force, venom

## Abstract

Historically, the role of aggression in the social lives of animals overwhelmingly focused on males. In recent years, however, female–female aggression in vertebrates, particularly lizards, has received increasing attention. This growing body of literature shows both similarities and differences to aggressive behaviours between males. Here, we document female–female aggression in captive Gila monsters (*Heloderma suspectum*). Based on four unique dyadic trials (eight adult female subjects), we developed a qualitative ethogram. Unexpected and most intriguing were the prevalence and intensity of aggressive acts that included brief and sustained biting, envenomation, and lateral rotation (i.e. rolling of body while holding onto opponent with closed jaws). Given specific behavioural acts (i.e. biting) and the results of bite-force experiments, we postulate that osteoderms (bony deposits in the skin) offer some degree of protection and reduce the likelihood of serious injury during female–female fights. Male–male contests in *H. suspectum,* in contrast, are more ritualized, and biting is rarely reported. Female–female aggression in other lizards has a role in territoriality, courtship tactics, and nest and offspring guarding. Future behavioural research on aggression in female Gila monsters is warranted to test these and other hypotheses in the laboratory and field.

## 1. Introduction

Aggression in animals has a long history of exploration and thus has been extensively studied in a wide assemblage of species, from the tiniest insects to the largest mammals [1–9]. In nearly all aspects of this research, emphasis was on males, which is understandable given that Darwin's monumental contributions to understanding antagonistic behaviour focused on males [1,2]. The evolutionary and ecological significance of aggression in conspecific males, for instance, has been extensively modelled in studies of territorial dominance, sexual selection, and mating systems [10–13]. Furthermore, a robust foundation of knowledge has been established on proximate mechanisms underlying the control and regulation of male aggression [7,8,14].

Females of many species, however, also engage in aggressive acts with each other, though compared with males the importance of these behaviours has received far less attention, and little is known regarding proximate mechanisms and ultimate causation [6,15–20]. Fortunately, this early trend, which largely deflected the significance of aggression in female vertebrates, is changing rapidly as new and exciting research has emerged over the past several decades. Lizards, in particular, have emerged as robust model systems for the study of female aggression, in part because many species are common and easily observed in nature or do well in laboratory or semi-captive conditions owing to their manageable size and ease of care [5,21–30].

Often associated with intrasexual aggression in animals, whether it be between males or between females, is the evolution of protective structures that reduce the likelihood of incurring injuries during fights. For example, the chest shield of male elephant seals [31] and the frill of *Triceratops* dinosaurs [32] are specialized structures that mitigate the potential for physical damage that could have fitness consequences. Although such morphologies are most apparent and widespread in relation to antagonistic behaviour by males, they similarly should be expected to occur and function in the context of female aggression and fighting.

The Gila monster (*Heloderma suspectum*) and closely related beaded lizards are large-bodied North American species that are notable for being venomous [33–38]. In addition, like many other reptiles (e.g. crocodilians, turtles, many lizards), Gila monsters have well-developed osteoderms (bony deposits in the skin) that appear well-suited to serve a protective function [33,36,39–42]. In nature, both sexes of *H. suspectum* have large home ranges, can be widely dispersed and travel considerable distances, and are likely to encounter each other during the mating season, especially in dense populations (reviewed in [36,43]). However, to the best of our knowledge, territoriality has not been formally tested in either sex of *H. suspectum*. Male–male agonistic contests have been documented in wild-living and captive *H. suspectum*; male–male interactions are ritualized and biting is rarely observed [36,44]. The occurrence and characteristics of aggression between females of this species, however, remains undocumented.

In this note, we describe female–female aggression in a captive colony of adult *H. suspectum* based on a sample of four unique staged interactions involving eight adult females. We combine our behavioural description with data from bite-force experiments to evaluate the prospect that the osteoderms of these lizards mitigate damage incurred from biting during aggressive interactions. We postulate that the protective function of the osteoderms, which are particularly well developed in the head and neck region, is potentially significant during escalated interactions between females.

## 2. Methods

### 2.1. Subjects and husbandry

A captive colony of 17 adult *H. suspectum* (9♂ : 8♀), originally obtained from zoological institutions and privately owned and maintained by one of us (K.H.P.), served as the source of subjects used in the behavioural study of female–female aggression. A separate colony of four adult *H. suspectum* (2♂ : 2♀), maintained in the Cal Poly Vivarium (California State Polytechnic University, Pomona, California), were used for bite-force experiments. For details of husbandry and sex determination, see electronic supplemental material, information, S1.

### 2.2. Behavioural trials

Female subjects were tested from 10.00 to 15.00 on 8–9 May, 1998 (Houston, Texas). This falls within the mating season of wild *H. suspectum*, which is from late April to early June, with egg-laying primarily occurring in late June and early July [45]. Two trials were run on each of the two days, with two unique subjects used in each trial, for a total of four trials. Ambient and substrate temperature during testing was 27–30°C, within the active temperature range (24.3–33.7°C) of this species ([33], see [36], p. 65, table 9).

Trials took place in a large circular galvanized steel stock tank (1.83 m diameter × 0.61 m high) with a substrate of decomposed granite to mimic natural material. A trial was initiated by simultaneously placing two adult female Gila monsters into opposite sides of the testing arena so that they were as far apart as possible and facing in opposite directions. The subjects were allowed to explore the arena without interruption. In all four trials, observations were made by two observers positioned approximately 2 m from the arena to avoid disturbing the animals. The arena was washed with detergent and the substrate changed prior to each trial to eliminate possible chemical cues from subjects in previous trials.

### 2.3. Bite-force experiments and morphometrics

Bite force was measured using a piezoelectric force transducer (type 9203, Kistler, Switzerland) custom fitted with stainless steel bite plates and connected to a charge amplifier (type 5995, Kistler, Switzerland) [46–48]. The transducer was prepared by adhering 5 mm thick leather pads at the ends of the bite plates where the lizards were induced to bite. The leather served to ensure that bites were applied at a consistent area along the bite plates, protect the lizards' teeth and avoid the potential for reduced performance that could be caused by biting on an unnatural hard surface (i.e. steel) [48]. The transducer was calibrated by hanging a series of weights with fishing line across the center of the leather pad on the bite plate that transmits forces to the Kistler transducer during bites.

**Figure 1.**
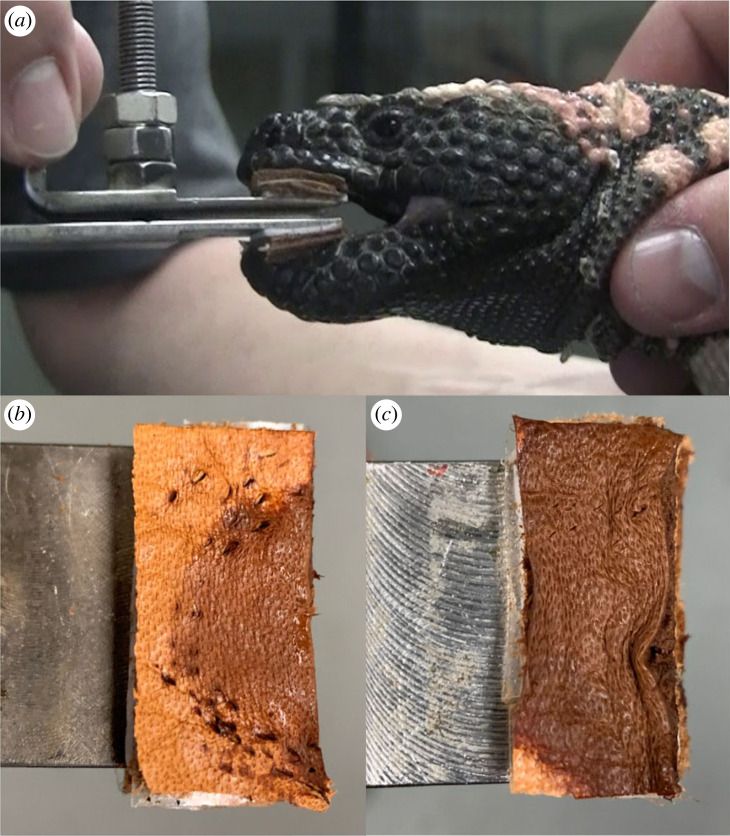
(*a*) Adult male *Heloderma suspectum* (CPP-66) biting force transducer in lateral view. (*b,c*) Close-up views of leather pads adhered to bite plates of force transducer. Note marks made by teeth that are most apparent on the leather pad on the upper bar (*b*). Copious amounts of venom-containing saliva conducted by the teeth of the lower jaw soaked the leather pad on the lower bar (*c*), causing the leather to swell and obscure the marks made by those teeth. By contrast to venomous snakes that have venom glands and venom-conducting teeth associated with the upper jaw, the venom glands of *H. suspectum* are located in the lower jaw with the venom-containing saliva conducted primarily by the lower teeth [33,36].

Prior to bite-force experiments, the subjects were put into a warm room (each in a separate plastic tub) for several hours so that all would have a similar body temperature. Body temperature during the experiments, measured by inserting a thermistor into the cloaca, was 24.7 ± 0.2°C (mean ± s.e.m.). Five bite-force trials, which were video-recorded from a lateral view (figure 1*a*), were performed on each lizard with 1 min of rest between trials. Gape angle was consistent among individuals (mean ± s.e.m. = 14.4 ± 0.18°) as measured from video frames across all trials using the angle function in NIH ImageJ v. 1.53k. Following previous studies [48], we standardized for variation in the bite position along the jaw line to compute bite force at two bite points, anterior bite force at the tips of the jaws and posterior bite force at the location of the most posterior teeth (approx. 65% of the distance from the quadrate-articular jaw joint to the jaw tips in *H. suspectum*). These two bite points encompass the possible range of bite out-levers, and therefore potential bite forces, during aggressive biting. For each individual, we report the greatest standardized bite force at each bite point.

To quantify body size, we measured body mass to the nearest 1 g with a digital scale (Taylor, model 39194CUS) and snout–vent length to the nearest 1 mm with a ruler. We quantified head size by measuring head length from the quadrate-articular jaw joint to the tip of the snout, maximum head width and maximum head depth to the nearest 0.1 mm using calipers [49].

## 3. Results

### 3.1. Behavioural trials

Aggressive behaviour involving eight different adult female subjects was documented in all four trials. Each trial was set to last 30 min, but three of four trials were under 15 min, as intervention was required to avoid potentially serious injury (e.g. see lateral rotation in table 1).

**Table 1.** Ethogram of 12 aggressive acts in staged, dyadic interactions between adult female *Heloderma suspectum*.

1. Tongue flicking (TF). When a trial is initiated, one subject typically approaches its opponent and exhibits TF. Soon thereafter both subjects exhibit rapid TF on the substrate and eventually on each other (i.e. TF with body contact).
2. Nudging (Nd). One subject, while performing TF, pushes its head laterally against the other female's head or another region of its anterior body.
3. Scratching (Sc). One subject exhibits Sc of its opponent using its front limbs. Sc can occur at any point following Nd.
4. Body Inflation (BI). Following Nd and sometimes Sc, one or both females may exhibit BI.
5. Hissing (Hs). Hissing often was coupled with BI, and it tends to be exaggerated when compared with Hs that occurs during courtship [K.H.P. 1993–2005, unpublished data]. Two types of Hs were exhibited: short bursts at relatively low volume versus extended and continuous vocalizations at relatively higher volume.
6. Chase-flee (CF). CF occurs when one subject chases the other as it flees.
7. Mounting (Mt). One female, typically the dominant individual, partially mounts or completely mounts (i.e. climbs on top of) its opponent. Other acts that were observed during Mt included TF, BI and Hs.
8. Holding (Hd). A female in the Mt position uses its front and back legs to grasp its opponent's torso and to maintain its Mt position.
9. Brief biting (BB). BB is when one female quickly bites or ‘nips' its opponent without holding on with its jaws.
10. Sustained biting (SB). SB is a prolonged bite with chewing-like motions by one female on the other.
11. Envenomation (En). En typically occurred during SB with repeated clamping of the closed jaws at a specific location on the opponent's body. En was apparent as oral secretions at the location of the bite, together with pronounced bleeding.
12. Lateral rotation (LR). LR is a spinning manoeuvre that involves rapid rotation of the body around its longitudinal axis. During LR, the forelimbs are adpressed on the venter, and the hindlimbs are outstretched and pressed along the tail.

### 3.2. Description of female aggressive behaviour

We provide a qualitative and generalized sequence of aggressive acts documented during the female–female dyadic interactions. We identified and describe 12 acts used during the interactions, from least intense (tongue-flicking) to most intense (lateral rotation) (table 1).

### 3.3. Bite force and morphometrics

The four Gila monsters that we tested bit the transducer vigorously. Typically, after the initial bite, the subject would maintain its grip on the transducer and repeatedly contract its jaw muscles. Following the trials for a given individual, salivary secretions were obvious on the leather pads adhered to the transducer bite plates (figure 1*b,c*), particularly the pad on the lower plate reflecting the ventral location of the venom-conducting teeth (figure 1*c*).

For the entire sample (*N* = 4 adult subjects), standardized anterior bite force at the tips of the jaws ranged from 35.7 to 94.3 N, and standardized posterior bite force at the most posterior teeth ranged from 55.0 to 145.1 N. Both of the males bit with greater force than both of the females (table 2). For all measures of body and head size, both of the males were larger than both of the females.

**Table 2.** Bite-force performance and morphometrics of *Heloderma suspectum*.

variable	female 1 (CPP-224)	female 2 (CPP-225)	male 1 (CPP-63)	male 2 (CPP-66)	mean ± s.e.m.
bite force—anterior (N)	35.7	59.9	80.5	94.3	67.6 ± 12.8
bite force—posterior (N)	55.0	92.1	123.9	145.1	104.0 ± 19.6
body mass (g)	423	526	581	659	625 ± 29.5
snout–vent length (mm)	300	294	325	336	313.8 ± 10.0
head length (mm)	47.0	47.1	51.6	56.8	50.6 ± 2.3
head width (mm)	44.0	49.5	50.7	56.4	50.2 ± 2.5
head depth (mm)	25.1	27.1	28.3	29.4	27.5 ± 0.9

## 4. Discussion

Based on a sample of four unique staged interactions involving eight subjects, we have documented unequivocally the occurrence of female–female aggression in a laboratory colony of the Gila monster (*H. suspectum*) and, in this note, provide a qualitative ethogram of behavioural acts. Based on earlier observations of adult female *H. suspectum* made by one of us (K.H.P.), aggression between females was not unexpected, though the complexity and intensity of acts we describe were largely unanticipated. Our observations of aggression between female *H. suspectum* are further supported by studies on female–female aggression in other lineages of lizards (e.g. [24,25] (Phrynosomatidae); [26] (Iguanidae); [28] (Scincidae); [50] (Varanidae); [51] (Dactyloidae); female aggression in reptiles reviewed in [52]). Female–female aggression also is present in the tuatara, *Sphenodon punctatus* [53,54], the sole extant representative of the ancient sister lineage to Squamata [55–57].

Our qualitative ethogram is the first description of acts used by Gila monsters during female–female interactions (table 1). Listed from least to most intense, we provide additional context for these behaviours. We presume tongue-flicking (TF) is a chemosensory act and is performed to ascertain sex and reproductive status of the other lizard [58,59]. In other species, TF also is done to assess and identify relatedness, such as in kin recognition [52,60]. In the four trials, all subjects continuously exhibited TF. We suspect nudging (Nd) and scratching (Sc) may signal to the opponent a willingness to engage in aggression. Sc was most evident when one female was mounted (Mt) on its opponent. During Nd and Sc, body inflation (BI) and hissing (Hs) were exhibited, and Hs often was coupled with BI. During female–female aggressive interactions, the body is inflated to a greater degree than we have observed by females during male–female courtship, but to a similar degree that males inflate their body during male–male contests (Gordon W. Schuett 1993–2005, personal observation). BI, exhibited throughout the staged interactions, may function as a visual display to increase perceived body size [36]. In a wide range of vertebrates, from fishes to great apes, BI and/or Hs are common in aggressive and defensive contexts [7,61]. Chase-Flee (CF) indicates the formation of a dominant–subordinate relationship; it also may indicate the end of a dyadic contest [62,63]. More aggressive subjects often would attempt to mount (Mt) their opponent, which we view as an act of dominance [7,61]. A female that had successfully mounted its opponent would sometimes hold (Hd) it tightly by partially embracing it around its torso, and the mounted female often would attempt to dislodge itself with rapid jerks and scything movements. When fights escalated, brief biting (BB) was often exhibited by both females concomitantly and occurred on any region of the body, including the head and limbs [7,36,61]. With further escalation, sustained biting (SB) occurred, followed by lateral rotation (LR). LR resembles the so-called ‘death roll’ of crocodilians ([64,65]; www.youtube.com/watch?v=xztDR4va4kE). LR is most often exhibited by an individual biting (SB) a limb of its opponent, though it also can be exhibited by the individual being bitten, typically together with rapid writhing movements.

We immediately stopped a trial when LR was exhibited to prevent potential injury. Grabbing each female generally resulted in a quick release. In one case, both subjects (the biter and the one being bitten) exhibited LR. During the intense acts of SB and LR, envenomation (En) occurred. Edema of the bitten region (most obvious when on a limb) typically occurred and pronounced lethargy was obvious for *ca* 24 h, probably from envenomation (see behavioural and physiological effects of *H*. *suspectum* envenomation in humans and other mammals; [36], pp. 44–52). There is a growing literature on venom (toxin) self-resistance in animals, including reptiles [66,67]. Despite limited information on self-resistance in *H. suspectum* and other helodermatid species [36,68,69], envenomated subjects in the present study were noticeably compromised both behaviourally and physically for a lengthy period, though recovery was full and without need for medical intervention.

In all four trials, we were surprised by how rapidly females initiated aggressive acts towards each other. The observations of SS, En and LR are particularly noteworthy; without our intervention to terminate three of the four trials, serious damage could have been inflicted. In all cases, one female of each dyad was the primary receiver of these dominant acts. Although no female Gila monster was permanently injured in the trials, under wild conditions this might not be the outcome of such interactions, particularly with respect to a combination of SB/En with LR. In crocodilians, although lateral rotation is often used to dismember prey, its primary function has been hypothesized as a means of escape or to injure an opponent during fights [65]. We propose that examining museum specimens of *H. suspectum* might reveal past injuries (e.g. broken long bones, dislocated joints, skull damage), and if those injuries are more common in females, this would suggest that such injuries can be incurred during fights between females.

By contrast to contests between adult male *H. suspectum*, which we have witnessed no fewer than 20 times in captivity (Karl H. Peterson and Gordon W. Schuett 1993–2005, unpublished data) and multiple times in the field (at least events we identified as male–male fights; see [70]), female–female fights appear to involve less ritualization. In fact, we have never observed SS, En or LR in encounters between male *H. suspectum*. Rather, it appears that male *H. suspectum* tend to exhibit repetitive acts that may serve to test the endurance of opponents, thus exhausting them to achieve dominance, especially when equally matched in size [36,44,71]. This is similar to male–male contests in certain lineages of venomous snakes, such as vipers, where biting is rarely reported [63,72]. In contests involving (putative) male beaded lizards (*H. horridum*), a large Neotropical congener of *H. suspectum*, biting has been reported to occur more frequently than in Gila monsters [73]. In other species of lizards [51] and the tuatara [54], females tend to show fewer agonistic displays than males and attack opponents more quickly in response to intrusions.

Aggression by females is expressed under multiple contexts and can profoundly influence their fitness [74]. Female–female aggressive behaviour in lizards can be related to acquisition and defence of territories, mate defence and parental care (e.g. defence of nest sites and progeny) [29,30,75–78]. In the tuatara, female–female aggression may have evolved in the context of nest defence [54]. Female–female aggression in *H. suspectum* may be related to one or more of these factors. In addition, the fact that the Gila monster is a nest-raiding specialist (primary diet of eggs, nestling birds and neonatal mammals; [36,79]) raises the intriguing possibility that aggression by nesting female *H. suspectum* toward marauding conspecifics, including other females, may function to prevent nest cannibalism. Although this study did not investigate the function of female–female aggression, which would require additional detailed information (e.g. documentation of post-oviposition nest presence, comparison of aggressiveness during different reproductive states), future behavioural research on intrasexual and potential intersexual aggression by female Gila monsters is warranted to test these and other hypotheses in the laboratory and field.

**Figure 2.**
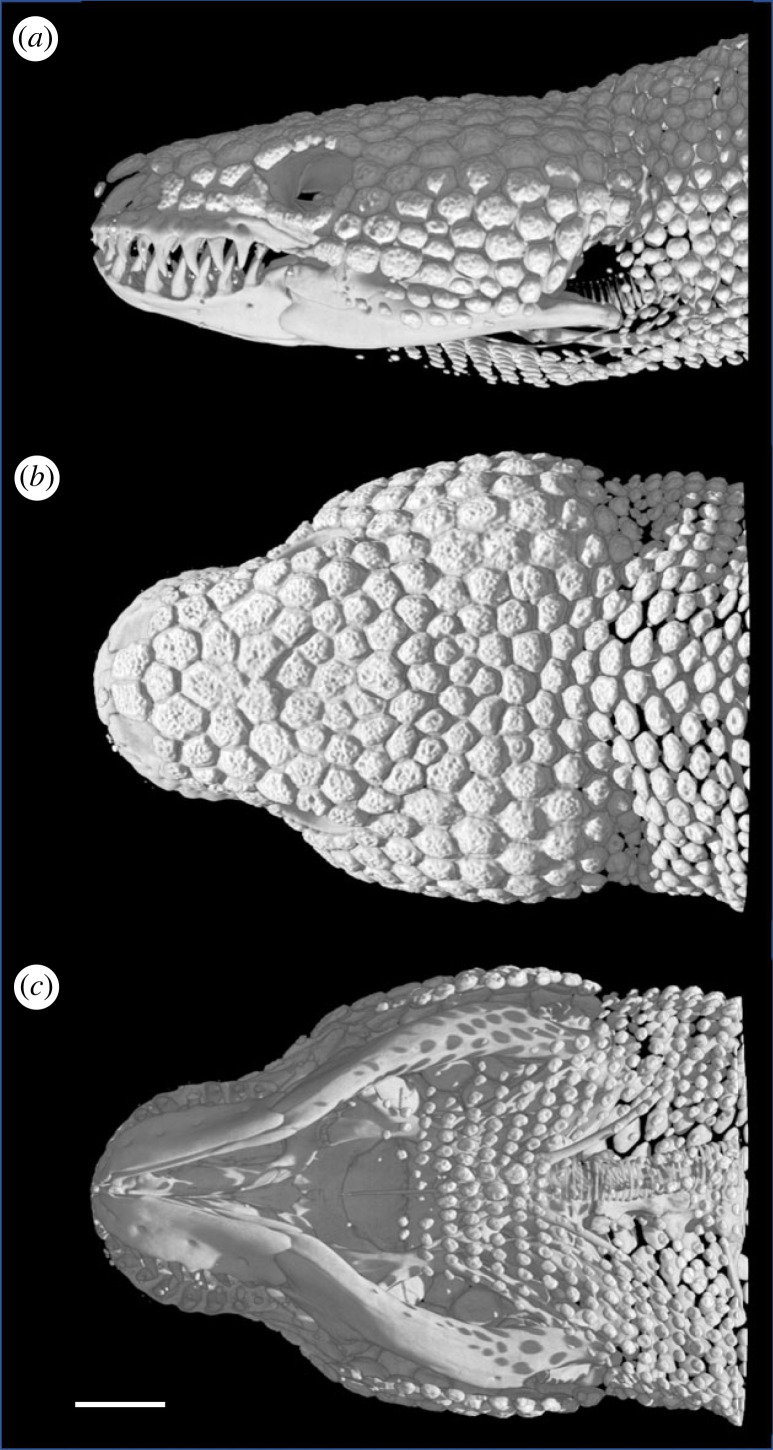
Osteoderms of the Gila monster. CT scans of the (*a*) lateral, (*b*) dorsal and (*c*) ventral aspects of the head of an adult *H. suspectum*. The top and sides of the head are well protected by large, closely spaced osteoderms. Osteoderms in the posterior gular region are relatively few and small, and the lower jaw completely lacks them. Note the formidable teeth of these lizards (*a*). Scale bar = 1 cm. Images courtesy of DigiMorph.com.

The dentition of helodermatid lizards comprises large, long, sharp and relatively few teeth, including those for conducting venom-containing saliva (figure 2*a*) [33,36]. This morphology is well-suited to achieve deep penetration during forceful biting, produce physical damage (e.g. to the integument), and effect envenomation efficacy [36]. As a nest-raiding specialist that consumes eggs and relatively helpless hatchlings/neonates, the primary diet of the Gila monster suggests that its impressive dentition is not strictly related to subjugating such prey; rather, it could possibly reflect an evolutionary history of predatory behaviour for which it was originally suited [36]. Furthermore, the teeth and venom of helodermatids play a role in defence against direct predators [36] and, as reported here, during conspecific fights between females. The potential for inflicting serious damage during a fight, combined with a protective function for osteoderms, suggests a possible reciprocal selective influence between the traits.

**Figure 3.**
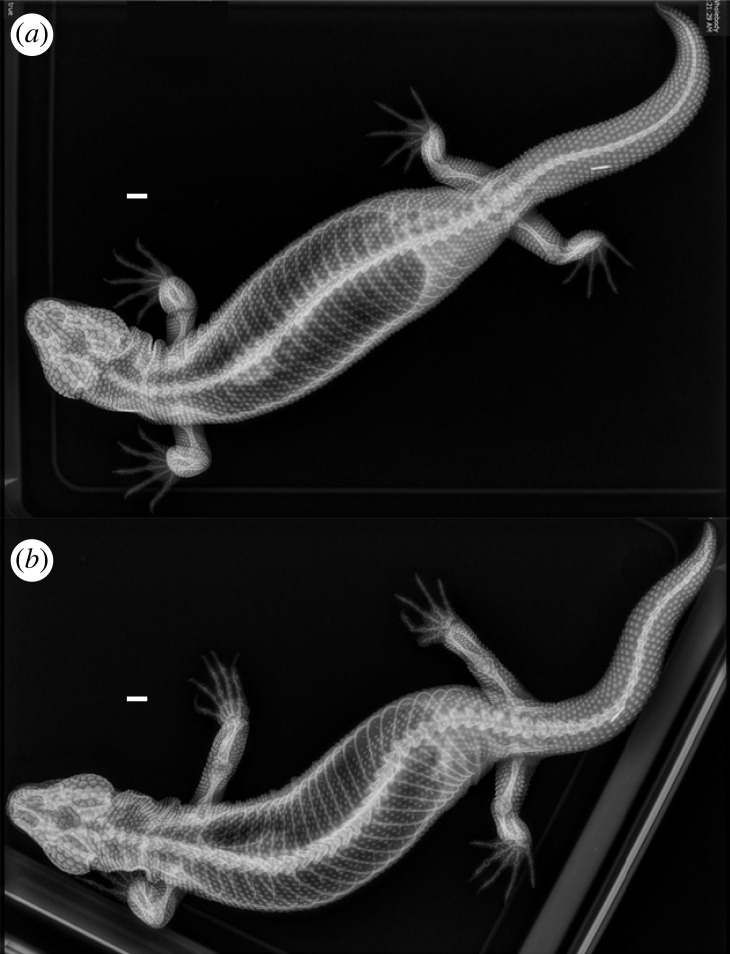
Osteoderms of the Gila monster. Whole body X-rays of (*a*) female (CPP-225) and (*b*) male (CPP-63) *H. suspectum* showing regional variation in osteoderm size and spacing of osteoderms (white dots). The largest and most closely spaced osteoderms are located on the head. Scale bars = 1 cm.

In all extant and extinct helodermatid lizards, osteoderms are well-developed and prevalent on the head, neck, limbs and dorsal torso of adults (figure 2, figure 3) [33,36]. As in other vertebrates, recent studies of the anatomical and functional properties of osteoderms in *H. suspectum* [40–42] provide experimental evidence that they can provide protection from predators [33,36]. Similarly, we suggest that osteoderms protect against potential damage that could be incurred during fights with conspecifics. Based on our measurements of bite-force performance, both female and male *H. suspectum* are capable of inflicting strong bites. The males in our sample bit considerably harder than the females, reflecting their greater body size and larger heads. If the results from our sample represent a general pattern, the greater capacity for forceful bites by males may reflect an undescribed role for biting by males of this species. Clearly, however, adult female *H. suspectum* can generate bite forces sufficient to penetrate the integument of a conspecific to the degree that bleeding and envenomation occurs, as was observed in our behavioural trials.

The osteoderms of *H. suspectum* are relatively large, and each one has a thick cap of highly mineralized tissue known as osteodermine [41]. The osteoderms are by far largest and most closely packed on the dorsal and lateral aspects of the head (figures 2 and 3), where a serious bite and/or envenomation probably would be the most detrimental. The neck also is well-protected by somewhat smaller osteoderms that remain quite closely spaced. The limbs have even smaller osteoderms associated with the smaller scales, although they are still fairly closely spaced thus providing some protection. The dorsal trunk has the least coverage by mineralized tissue of any region containing osteoderms, with small osteoderms that are the most widely spaced of any on the body, and the tail similarly has only modest coverage. Although large osteoderms that lack gaps between them would provide the best protection during female–female aggression, the presence of osteoderms of any size and arrangement probably provide some protection. During a bite, many teeth simultaneously engage the integument of the opponent. Even if only a few teeth engage osteoderms, the pressure applied by adjacent teeth (not engaged with osteoderms) would be reduced and thus mitigate overall tissue damage. Interestingly, the ventrolateral and ventral surfaces of the torso completely lack protection via osteoderms [33,80], perhaps because these areas tend to be more protected by the substrate. Body inflation during fights may compensate for this deficiency by making it more difficult to grip an opponent with the jaws in these regions. Whether or not osteoderms show sexual dimorphism in *H. suspectum* and other helodermatids has yet to be reported [80], though our preliminary X-ray imaging reveals no obvious differences in size, number, or location.

The fact that male–male agonistic interactions in *H. suspectum* are more ritualized than those between females may be related to an interplay between the capacity to inflict damaging bites, both with respect to mechanical injury and venom conduction, and a protective function of osteoderms. Although osteoderms should provide some protection for both sexes, a greater capacity for strong biting by males indicates that they nevertheless should be capable of inflicting more damage than females. This reduces the effectiveness of osteoderms in mitigating damage if males were to bite each other in the intense manner observed during female–female interactions. The literature (e.g. [36,44,71,81]) and personal observations by G.W.S. indicate that male–male contests in Gila monsters are less violent than our present description of female–female fights (i.e. male contests lack sustained biting, envenomation, lateral rotation). This suggests that ritualized contests in male *H. suspectum* may have evolved to reduce the likelihood of serious injury to both participants during agonistic intrasexual interactions, a common pattern in the social behaviour of animals that has theoretical and empirical support [63,82–84].

In conclusion, though our study was limited to a small sample of subjects, it provides clear and important directions for future research on aggression in Gila monsters, especially with respect to female–female interactions. We suggest that additional laboratory research should (i) test a larger sample of female subjects using a formal design for trials, (ii) include videographic and quantitative analyses of aggressive acts, (iii) test both sexes, including trials in which females are paired with males, and (iv) incorporate variables such as season and reproductive status to examine their effects on social behaviour.

## Data Availability

Data are available in the Dryad Digital Repository: https://doi.org/10.5061/dryad.bnzs7h4fd [85]. Supplementary material is available online [86].

## References

[1] Darwin C. 1859 On the origin of species by means of natural selection. London, UK: John Murray.

[2] Darwin C. 1871 The descent of man, and selection in relation to sex. London, UK: John Murray.

[3] Lorenz K. 1966 On aggression. London, UK: Methuen.

[4] Wilson EO. 1971 The insect societies. Cambridge, MA: Harvard University Press.

[5] Stamps JA. 1977 The relationship between resource competition, risk, and aggression in a tropical territorial lizard. Ecology **58**, 349-358. (10.2307/1935609)

[6] Bekoff M. 1981 Development of agonistic behaviour: ethological and ecological aspects. In Multidisciplinary approaches to aggression research (eds PF Brain, D Benton), pp. 161-178. New York, NY: Elsevier/North Holland Biomedical Press.

[7] Archer J. 1988 The behavioural biology of aggression. Cambridge, UK: Cambridge University Press.

[8] Hardy IC, Briffa M. 2013 Animal contests. Cambridge, UK: Cambridge University Press.

[9] Tibbetts EA, Pardo-Sanchez J, Weise C. 2022 The establishment and maintenance of dominance hierarchies. Phil. Trans. R. Soc. B **377**, 20200450. (10.1098/rstb.2020.0450) 35000449 PMC8743888

[10] Maynard Smith J. 1982 Evolution and the theory of games. Cambridge, UK: Cambridge University Press. http://www.jstor.org/stable/27847040?origin=JSTOR-pdf

[11] Sinervo B, Lively CM. 1996 The rock-paper-scissors game and the evolution of alternative male strategies. Nature **380**, 240-243. (10.1038/380240a0)

[12] Hsu Y, Earley RL, Wolf LL. 2006 Modulation of aggressive behaviour by fighting experience: mechanisms and contest outcomes. Biol. Rev. **81**, 33-74. (10.1017/S146479310500686X) 16460581

[13] Dugatkin LA. 2020 Principles of animal behaviour. Chicago, IL: University of Chicago Press.

[14] Moore MC, Lindzey J. 1992 The physiological basis of sexual behaviour in male reptiles. In Biology of the reptilia, vol. 18 (eds C Gans, D Crews), pp. 70-113. Chicago, IL: The University of Chicago Press.

[15] Andrews TJ, Summers CH. 1996 Aggression, and the acquisition and function of social dominance in female *Anolis carolinensis*. Behaviour **133**, 1265-1279. (10.1163/156853996×00396)

[16] Earley RL, Attum O, Eason P. 2002 Varanid combat: perspectives from game theory. Amphibia-Reptilia **23**, 469-485. (10.1163/15685380260462374)

[17] Prosen ED, Jaeger RG, Lee DR. 2004 Sexual coercion in a territorial salamander: females punish socially polygynous male partners. Anim. Behav. **67**, 85-92. (10.1016/j.anbehav.2003.02.005)

[18] Filby AL, Paul GC, Hickmore TFA, Tyler CR. 2010 Unravelling the neurophysiological basis of aggression in a fish model. BMC Genomics **11**, 498. (10.1186/1471-2164-11-498) 20846403 PMC2996994

[19] Duque-Wilckens N, Trainor BC. 2017 Behavioral neuroendocrinology of female aggression. Oxford Res. Encycl. Neurosc. (10.1093/acrefore/9780190264086.013.11)

[20] Zubizarreta L, Silva AC, Quintana L. 2020 The estrogenic pathway modulates non-breeding female aggression in a teleost fish. Physiol. Behav. **220**, 112883. (10.1016/j.physbeh.2020.112883) 32199998

[21] Simon CA. 1975 The influence of food abundance on territory size in the iguanid lizard *Sceloporus jarrovi*. Ecology **56**, 993-999. (10.2307/1936311)

[22] Warwick C, Frye FL, Murphy JB. 1995 Health and welfare of captive reptiles. London, UK: Chapman & Hall.

[23] Summers TR, Hunter AL, Summers CH. 1997 Female social reproductive roles affect central monoamines. Brain Res. **767**, 272-278. (10.1016/S0006-8993(97)00604-5) 9367258

[24] Woodley SK, Moore MC. 1999 Female territorial aggression and steroid hormones in mountain spiny lizards. Anim. Behav. **57**, 1083-1089. (10.1006/anbe.1998.1080) 10328794

[25] Hews DK, Castellano MJ, Hara E. 2004 Aggression in females is also lateralized: left-eye bias during aggressive courtship rejection in lizards. Anim. Behav. **68**, 1201-1207. (10.1016/j.anbehav.2003.11.024)

[26] Rubenstein D, Wikelski M. 2005 Steroid hormones and aggression in female Galápagos marine iguanas. Horm. Behav. **48**, 329-341. (10.1016/j.yhbeh.2005.04.006) 15916763

[27] Kabelik D, Weiss SL, Moore MC. 2008 Steroid hormones alter neuroanatomy and aggression independently in the tree lizard. Physiol. Behav. **93**, 492-503. (10.1016/j.physbeh.2007.10.008) 17996258 PMC4286361

[28] Sinn DL, While GM, Wapstra E. 2008 Maternal care in a social lizard: links between female aggression and offspring fitness. Anim. Behav. **76**, 1249-1257. (10.1016/j.anbehav.2008.06.009)

[29] Sherbrooke WC. 2017 Antipredator nest guarding by female horned lizards (*Phrynosoma*): iguanian parental care. Herpetologica **73**, 331-337. (10.1655/Herpetologica-D-17-00028.1)

[30] Wu Y, Ramos JA, Qiu X, Peters RA, Qi Y. 2018 Female–female aggression functions in mate defence in an Asian agamid lizard. Anim. Behav. **135**, 215-222. (10.1016/j.anbehav.2017.11.023)

[31] Leboeuf BJ. 1972 Sexual behavior in the northern elephant seal *Mirounga angustirostris*. Behaviour **41**, 1-26. (10.1163/156853972x00167) 5062032

[32] Farke AA, Wolff EDS, Tanke DH. 2009 Evidence of combat in *Triceratops*. PLoS ONE **4**, e4252. (10.1371/journal.pone.0004252) 19172995 PMC2617760

[33] Bogert CM, Martín del Campo R. 1956 The Gila monster and its allies: the relationships, habits, and behaviour of the lizards of the family Helodermatidae. Bull. Am. Mus. Nat. Hist. **109**, 1-238. (10.2307/1446741)

[34] Beck DD, Lowe CH. 1991 Ecology of the beaded lizard, *Heloderma horridum*, in a tropical dry forest in Jalisco, México. J. Herpetol. **25**, 395-406. (10.2307/1564760)

[35] Campbell JA, Lamar WW. 2004 The venomous reptiles of the Western Hemisphere, vols. 1 and 2. Ithaca, NY: Cornell University Press.

[36] Beck DD. 2005 Biology of Gila monsters and beaded lizards. Berkeley, CA: University of California Press.

[37] Douglas ME, Douglas MR, Schuett GW, Beck DD, Sullivan BK. 2010 Conservation phylogenetics of helodermatid lizards using multiple molecular markers and a supertree approach. Mol. Phylogenet. Evol. **55**, 153-167. (10.1016/j.ympev.2009.12.009) 20006722

[38] Reiserer RS, Schuett GW, Beck DD. 2013 Taxonomic reassessment and conservation status of the beaded lizard, *Heloderma horridum* (Squamata: Helodermatidae). Amphib. Rept. Conserv. **7**, 74-96.

[39] Pregill GK, Gauthier JA, Greene HW. 1986 The evolution of helodermatid squamates, with description of a new taxon and an overview of Varanoidea. Trans. San Diego Soc. Nat. Hist. **21**, 167-202.

[40] Iacoviello A et al. 2020 The multiscale hierarchical structure of *Heloderma suspectum* osteoderms and their mechanical properties. Acta Biomat. **107**, 194-203. (10.1016/j.actbio.2020.02.029) 32109598

[41] Kirby A, Vickaryous M, Boyde A, Olivo A, Moazen M, Bertazzo S, Evans S. 2020 A comparative histological study of the osteoderms in the lizards *Heloderma suspectum* (Squamata: Helodermatidae) and *Varanus komodoensis* (Squamata: Varanidae). J. Anat. **236**, 1035-1043. (10.1111/joa.13156) 31986227 PMC7219622

[42] Williams C et al. 2022 A review of the osteoderms of lizards (Reptilia: Squamata). Biol. Rev. **97**, 1-19. (10.1111/brv.12788) 34397141 PMC9292694

[43] Kwiatkowski MA, Schuett GW, Repp RA, Nowak EM, Sullivan BK. 2008 Does urbanization influence the spatial ecology of Gila monsters in the Sonoran Desert? J. Zoology **276**, 350-357. (10.1111/j.1469-7998.2008.00495.x)

[44] Beck DD. 1990 Ecology and behaviour of the Gila monster in southwestern Utah. J. Herpetol. **24**, 54-68. (10.2307/1564290)

[45] DeNardo DF, Moeller KT, Seward M, Repp R. 2018 Evidence for atypical nest overwintering by hatchling lizards, *Heloderma suspectum*. Proc. R. Soc. B **285**, 20180632. (10.1098/rspb.2018.0632) PMC5998102 29794051

[46] Herrel A, Spithoven L, Van Damme R, De Vree F. 1999 Sexual dimorphism of head size in *Gallotia galloti*: testing the niche divergence hypothesis by functional analyses. Funct. Ecol. **13**, 289-297. (10.1046/j.1365-2435.1999.00305.x)

[47] Lappin AK, Husak JF. 2005 Weapon performance, not size, determines mating success and potential reproductive output in the collared lizard (*Crotaphytus collaris*). Am. Nat. **166**, 426-436. (10.1086/432564) 16224696

[48] Lappin AK, Jones MEH. 2014 Reliable quantification of bite-force performance requires use of appropriate biting substrate and standardization of bite out-lever. J. Exp. Biol. **217**, 4303-4312. (10.1242/jeb.106385) 25359934

[49] Lappin AK, Hamilton PS, Sullivan BK. 2006 Bite-force performance and head shape in a sexually dimorphic crevice-dwelling lizard, the common chuckwalla [*Sauromalus ater* (= *obesus*)]. Biol. J. Linn. Soc. **88**, 215-222. (10.1111/j.1095-8312.2006.00615.x)

[50] King D, Green B. 1999 Goannas: the biology of varanid lizards, 2nd edn. Sydney, Australia: University of South Wales Press.

[51] Reedy AM, Pope BD, Kiriazis NM, Giordano CL, Sams CL, Warner DA, Cox RM. 2017 Female anoles display less but attack more quickly than males in response to territorial intrusions. Behav. Ecol. **28**, 1323-1328. (10.1093/beheco/arx095)

[52] Doody JS, Dinets V, Burghardt GM. 2021 The secret social lives of reptiles. Baltimore, MD: Johns Hopkins University Press.

[53] Refsnider JM, Keall SN, Daugherty CH, Nelson NJ. 2009 Does nest-guarding in female tuatara (*Sphenodon punctatus*) decrease nest destruction by conspecific females? J. Herpetol. **43**, 294-299. (10.1670/08-120R1.1)

[54] Ramstad KM, Moore JA, Refsnider JM. 2012 Intrasexual aggression in tuatara: males and females respond differently to same-sex intruders during mating season. Herpetol. Rev. **43**, 19-21.

[55] Gauthier JA, Kearney M, Maisano JA, Rieppel O, Behike ADB. 2012 Assembling the squamate tree of life: perspectives from the phenotype and the fossil record. Bull. Peabody Mus. Nat. Hist. **53**, 3-308. (10.3374/014.053.0101)

[56] Reeder TW, Townsend TM, Mulcahy DG, Noonan BP, Wood Jr PL, Sites Jr JW, Wiens JJ. 2015 Integrated analyses resolve conflicts over squamate reptile phylogeny and reveal unexpected placements for fossil taxa. PLoS ONE **10**, e0118199. (10.1371/journal.pone.0118199) 25803280 PMC4372529

[57] Burbrink FT et al. 2020 Interrogating genomic-scale data for Squamata (lizards, snakes, and amphisbaenians) shows no support for key traditional morphological relationships. Syst. Biol. **69**, 502-520. (10.1093/sysbio/syz062) 31550008

[58] Halpern M, Martinez-Marcos A. 2003 Structure and function of the vomeronasal system: an update. Prog. Neurobiol. **70**, 245-318. (10.1016/S0301-0082(03)00103-5) 12951145

[59] Mason RT, Parker MR. 2010 Social behaviour and pheromonal communication in reptiles. J. Comp. Physiol. A, Neuroethol., Sensory, Neural, and Behav. Physiol. **196**, 729-749. (10.1007/s00359-010-0551-3) 20585786

[60] Clark RW. 2004 Kin recognition in rattlesnakes. Biol. Lett. **271**, S243-S245. (10.1098/rsbl.2004.0162) PMC1810029 15252996

[61] Greene HW. 1988 Antipredator mechanisms in reptiles. In Biology of the reptilia, vol. 16 (eds C Gans, RB Huey), pp. 1-152. New York, NY: John C. Wiley and Sons.

[62] Dugatkin LA. 1997 Winner and loser effects and the structures of dominance hierarchies. Behav. Ecol. **8**, 583-587. (10.1093/beheco/8.6.583)

[63] Schuett GW. 1997 Body size and agonistic experience affect dominance and mating success in male copperheads. Anim. Behav. **54**, 213-224. (10.1006/anbe.1996.0417) 9268451

[64] Fish FE, Bostic SA, Nicastro AJ, Beneski JT. 2007 Death roll of the alligator: mechanics of twist feeding in water. J. Exp. Biol. **210**, 2811-2818. (10.1242/jeb.004267) 17690228

[65] Drumheller SK, Darlington J, Vliet KA. 2019 Surveying death roll behaviour across Crocodylia. Ethol. Ecol. Evol. **31**, 329-347. (10.1080/03949370.2019.1592231)

[66] Straight R, Glenn JL, Snyder CC. 1976 Antivenom activity of rattlesnake blood plasma. Nature **261**, 259-260. (10.1038/261259a0) 1272402

[67] van Thiel J et al. 2022 Convergent evolution of toxin resistance in animals. Biol. Rev. **97**, 1823-1843. (10.1111/brv.12865) 35580905 PMC9543476

[68] Loeb L et al. 1913 *The venom of* Heloderma, vol. 177, pp. 1-244. Washington, DC: Carnegie Institute of Washington.

[69] Woodson WW. 1947 Toxicity of *Heloderma* venom. Herpetologica **4**, 31-33.

[70] Zylstra ER, Seward MT, Schuett GW, Repp RA, DeNardo DF, Beck DD. 2015 Natural history notes. *Heloderma suspectum* (Gila monster): probable courtship and mating behaviour. Herpetol. Rev. **46**, 258-259.

[71] Gienger CM, Beck DD. 2007 Heads or tails? Sexual dimorphism in helodermatid lizards. Canad. J. Zool. **85**, 92-98. (10.1139/z06-198)

[72] Schuett GW, Gergus EWA, Kraus F. 2001 Phylogenetic correlation between male–male fighting and mode of prey subjugation in snakes. Acta Ethol. **4**, 31-49. (10.1007/s102110100043)

[73] Beck DD, Ramirez-Bautista A. 1991 Combat behaviour of the beaded lizard, *Heloderma h. horridum*, in Jalisco, Mexico. J. Herpetol. **25**, 481-484. (10.2307/1564773)

[74] Gill SA, Alfson ED, Hau M. 2007 Context matters: female aggression and testosterone in a year-round territorial Neotropical songbird (*Thryothorus leucotis*). Proc. R. Soc. B **274**, 2187-2194. (10.1098/rspb.2007.0457) PMC2706190 17609184

[75] Wiewandt TA. 1982 Evolution of nesting patterns in iguanine lizards. In Iguanas of the world: their behaviour, ecology, and conservation (eds GM Burghardt, AS Rand), pp. 119-141. Parkwood, NJ: Noyes Publications.

[76] Attum O, Earley RL, Bayless M, Eason P. 2000 The agonistic behaviour of Bosc's monitor (*Varanus exanthematicus* Bosc, 1792) in captivity. Herpetol. Bull. **73**, 22-26.

[77] Baird TA, Sloan SL. 2003 Interpopulation variation in the social organization of female collared lizards, *Crotaphytus collaris*. Ethology **109**, 879-894. (10.1046/j.0179-1613.2003.00925.x)

[78] Pike DA, Clark RW, Manica A, Tseng H-Y, Hsu J-Y, Huang W-S. 2016 Surf and turf: predation by egg-eating snakes has led to the evolution of parental care in a terrestrial lizard. Sci. Reps. **6**, 22207. (10.1038/srep22207) PMC4768160 26915464

[79] Repp RA, Schuett GW. 2009 Natural history notes. *Heloderma suspectum* (Gila monster): diet and predatory behavior. Herpetol. Rev. **40**, 343-345.

[80] Broeckhoven C, de Kock C, Hui C. 2018 Sexual dimorphism in the dermal armour of cordyline lizards (Squamata: Cordylinae). Biol. J. Linn. Soc. **125**, 30-36. (10.1093/biolinnean/bly096)

[81] Demeter BJ. 1986 Combat behavior in the Gila monster (*Heloderma suspectum cinctum*). Herpetol. Rev. **17**, 9-11.

[82] Crane J. 1966 Display and ritualization in fiddler crabs (Ocypodidae, genus *Uca*). Phil. Trans. R. Soc. B **251**, 459-472. (10.1098/rstb.1966.0035)

[83] Parker GA. 1974 Assessment strategy and the evolution of fighting behaviour. J. Theoretical Biol. **47**, 223-243. (10.1016/0022-5193(74)90111-8) 4477626

[84] West-Eberhard MJ. 1979 Sexual selection, social competition, and evolution. Proc. Amer. Phil. Soc. **123**, 222-234.

[85] Schuett GW, Peterson KH, Powell AR, Taylor JD, Alexander JR, Lappin AK. 2023 Data from: Female–female aggression in the Gila monster (*Heloderma suspectum*). Dryad Digital Repository. (10.5061/dryad.bnzs7h4fd) PMC10170349 37181791

[86] Schuett GW, Peterson KH, Powell AR, Taylor JD, Alexander JR, Lappin AK. 2023 Female–female aggression in the Gila monster (*Heloderma suspectum*). Figshare. (10.6084/m9.figshare.c.6619754) PMC10170349 37181791

